# Passive and Active Vaccination Strategies to Prevent Ricin Poisoning

**DOI:** 10.3390/toxins3091163

**Published:** 2011-09-15

**Authors:** Seth H. Pincus, Joan E. Smallshaw, Kejing Song, Jody Berry, Ellen S. Vitetta

**Affiliations:** 1 Children’s Hospital and LSU Health Sciences Center, New Orleans, LA 70118, USA; Email: spincus@chnola-research.orgs; 2 Cancer Immunobiology Center and Department of Microbiology, University of Texas, Southwestern Medical Center, Dallas, TX 75235, USA; Email: Joan.smallshaw@utsouthwestern.edu; 3 Children’s Hospital, New Orleans, LA 70118, USA; Email: ksong@chnola-research.org; 4 Cangene Corporation, Winnipeg, MB R3T 5Y3, Canada; Email: jberry@cangene.com; 5 Cancer Immunobiology Center, Departments Of Immunology and Microbiology, University of Texas Southwestern Medical Center, Dallas, TX 75230, USA

**Keywords:** ricin, biothreat, vaccines, antibodies

## Abstract

Ricin toxin (RT) is derived from castor beans, produced by the plant *Ricinus communis*. RT and its toxic A chain (RTA) have been used therapeutically to arm ligands that target disease-causing cells. In most cases these ligands are cell-binding monoclonal antibodies (MAbs). These ligand-toxin conjugates or immunotoxins (ITs) have shown success in clinical trials [1]. Ricin is also of concern in biodefense and has been classified by the CDC as a Class B biothreat. Virtually all reports of RT poisoning have been due to ingestion of castor beans, since they grow abundantly throughout the world and are readily available. RT is easily purified and stable, and is not difficult to weaponize. RT must be considered during any “white powder” incident and there have been documented cases of its use in espionage [2,3]. The clinical syndrome resulting from ricin intoxication is dependent upon the route of exposure. Countermeasures to prevent ricin poisoning are being developed and their use will depend upon whether military or civilian populations are at risk of exposure. In this review we will discuss ricin toxin, its cellular mode of action, the clinical syndromes that occur following exposure and the development of pre- and post-exposure approaches to prevent of intoxication.

## 1. Ricin Toxin

RT is a disulfide-linked heterodimeric glycoprotein consisting of the toxic RTA and the cell-binding B chain (RTB). RTA is an *N*-glycosidase that specifically cleaves the 28S ribosomal RNA at adenine 4324. RTB is a galactose-specific lectin, that binds to cell-surface glycolipids and glycoproteins found on all vertebrate cells. The toxin is extracted from castor beans, where it consists of 3–5% of the bean’s dry weight [2]. *Ricinus communis* grows worldwide in warm temperate and tropical climates. It is cultivated as an ornamental plant and used commercially for its oil; it also grows as a weed. Castor oil is extracted for its utility as a high temperature lubricant. Following the extraction of the oil, the toxin can be purified from the remaining mash by sodium hydroxide precipitation (crude preparation) or by chromatography. However, even crude RT is highly toxic; pulverized beans are a potential bioweapon. The purified toxin is a white powder, and is quite stable at temperatures below 60°. However, even when boiled, high does of RT can be lethal (Vitetta *et al.*, unpublished). The ubiquity of castor beans, ease of extraction, and chemical stability, make ricin an attractive and inexpensive agent for scientifically unsophisticated individuals or nations to produce in large quantities. The LD_50_ of ricin toxin varies according to the route of exposure, 5–15 µg/kg by aerosol or parenteral administration, 25–100 mg/kg orally [2,4,5] or 5–15 µg/kg by gastric gavage following a period of fasting [6]. While RT is 100-fold less toxic than botulinum toxin, even smaller scale events in civilian populations could lead to panic and economic disruption, which are the basic objectives of terrorism [7]. Given the history of RT-related events [2,3,7], there is no question that it will at some point be used in an act of terrorism. Indeed caches of RT have been found throughout the world [7] and in August, 2011 it was reported by the New York Times that Al-Qaeda was experimenting with ricin bombs.

## 2. Cellular Toxicity of RT

RT is a biochemically simple molecule, which must be routed to specific sites in the cell to exert its toxicity [8]. As a result, the processing of RT has been well studied and informative regarding unique trafficking pathways in the cell, notably “reverse transport”. There are several excellent review articles that describe the intracellular processing of RT [2,9,10,11,12,13]. This review will focus on those aspects of RT that can be targeted by antibodies or other inhibitors and the reader is referred to the cited reviews for biochemical details.

RTB binds to cells *via* its lectin receptors [9]. Because many cell surface glycoproteins and glycolipids display terminal galactosyl residues, RT binds promiscuously to virtually all cell types. Because each RT monomer can bind to two galactose-containing residues, RT can also cross-link some cell surface molecules. The binding of the toxin to cells is a target for intervention, by both antibodies and competitive ligands [14]. We routinely use 0.1 M lactose or galactose solutions to block the cellular cytotoxicity of RT *in vitro*. Milk contains a similar concentration of lactose, and thus we use the milk-based Blocker ^TM^ BLOTTO to block non-specific binding of glycoproteins to ricin in immunoassays. One wonders whether the antidote to ricin ingestion might not be a simple glass of milk, assuming it is given within a short period of time. To our knowledge this has not been investigated. It is also possible that microbial polysaccharides bind to RT at mucosal sites, and thus hinder its binding to human cells. This could account for the low toxicity of orally administered RT.

RT is internalized from the cell surface *via* a variety of mechanisms, both clathrin dependent and independent, and dynamin dependent and independent. By cross-linking cell surface molecules, and signaling through cell surface kinases, it is possible that RT can upregulate its own uptake into cells [9]. Because RT can bind to a number of cell surface glycan-containing structures, as well as glycoproteins in the serum and tissues, it is likely to be internalized by the full gamut of uptake mechanisms, including phagocytosis and micropinocytosis. Since the mechanisms of intake of RT differ in different cells as well as within the same cell, it can be deposited in different intracellular sites. 

**Figure 1:**
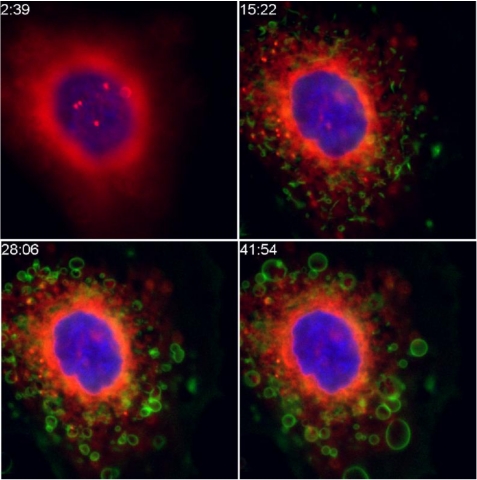
Expulsion of ricin from intoxicated cells. Cells were labeled with a nuclear dye (blue), and with Bodipy-brefeldin A, which labels ER and Golgi (red). Ricin (green) was added at 3 min post initiation of timing, shown as min:sec in the time series. Within 15 min the ricin has begun to colocalize with the Bodipy-BFA (showing orange). Individual ricin-coated blebs move outward from the cell, most clearly seen between the latter time points.

Much of the initial intracellular routing of RT involves shuttling among endosomal compartments. Ricin may follow three paths from the endosomes. The first involves exocytosis and expulsion from the cell, likely involving blebbing. Figure 1 shows micrographs of this process. The blebbing might indicate an attempt by the cell to expel the toxin. It might also represent a potential way for active ricin to be transferred to other cells in a tissue. The second pathway leads to lysosomal degradation. It is only by the third, retrograde, route that RT reaches its intracellular site of action. RT traverses the same set of organelles involved in the secretion of proteins, but does so in the reverse direction used by secreted proteins, moving from the endosomes, through the Golgi, and into the ER. Initially it was believed that this retrograde path was unique to toxins, but it has been found to be a regular feature of intracellular trafficking [9,10,13]. Drugs that specifically inhibit this pathway have been developed and have been show to be effective anti-RT agents, both in cells and in animals, albeit at very high concentrations [8].

Once RT reaches the endoplasmic reticulum, it must be translocated across the ER membrane into the cytosol, where it exerts its toxicity. Within the ER, the holotoxin is reduced, and RTA is released. The RTA then unfolds, crosses the ER membrane and translocates into the cytosol [15,16]. To successfully accomplish this, the unfolded A chain must avoid ubiquitination and the ER-associated degradation pathway, while still utilizing protein conducting translocons that are part of the degradation pathways. This is accomplished through the use of chaperones including Hsc70 and Hsp90 [16], as well as by a temperature-dependent structural alteration in RTA, involving the loss of alpha-helical structures and insertion of the *C*-terminus into the membrane lipid bilayer of the ER [15]. 

Once at its site of action, RTA acts as an *N*-glycosidase, depurinating ribosomal RNA by removing adenine 4324 in the RTA/sarcin loop of the 28S rRNA. The enzyme inactivates 1500 ribosomes per minute. The high catalytic rate has been found to be due, in part, to movement of the RNA structure itself [17]. It has been proposed that a helical domain of RTA, (amino acids 99–106) also plays a key role in depurination, and that the function of this domain might be hindered by an antibody (Ab)-mediated attack [18]. Chemical analogues that mimic RNA’s transition states during catalytic cleavage have been studied as potential inhibitors of RT [19,20,21,22]. 

It has been estimated that for each functional RT molecule that reaches its site of action, 10,000 other internalized molecules have either been degraded, eliminated by exocytosis, or sequestered in functionally irrelevant compartments in the cell [23]. Cell death results from inhibition of protein synthesis and is primarily by apoptosis.

## 3. Beneficial Uses of Ricin

The authors of this review first studied RT because of its therapeutic potential, primarily as the toxic moiety of MAb-based immunotoxins (ITs). ITs are part of a larger class of agents, termed immunoconjugates (ICs), that consist of a targeting moiety linked to a cytotoxic moiety. If the toxic moiety is a protein toxin or its active subunit, these ICs are called ITs. Projects under active investigation in our laboratories include ITs to treat lymphomas and leukemias [24,25,26], ITs as immunomodulatory agents [27], and ITs to eliminate the latent reservoir of HIV that remains following antiretroviral treatment [28,29]. The clinical efficacy of ITs and immunoconjugates has clearly been demonstrated in human clinical trials [30]. While there was initial concern regarding the widespread inherent toxicity of ITs, this was not the case; the major-dose limiting toxicity in early trials was vascular leak syndrome (VLS). 

**Figure 2:**
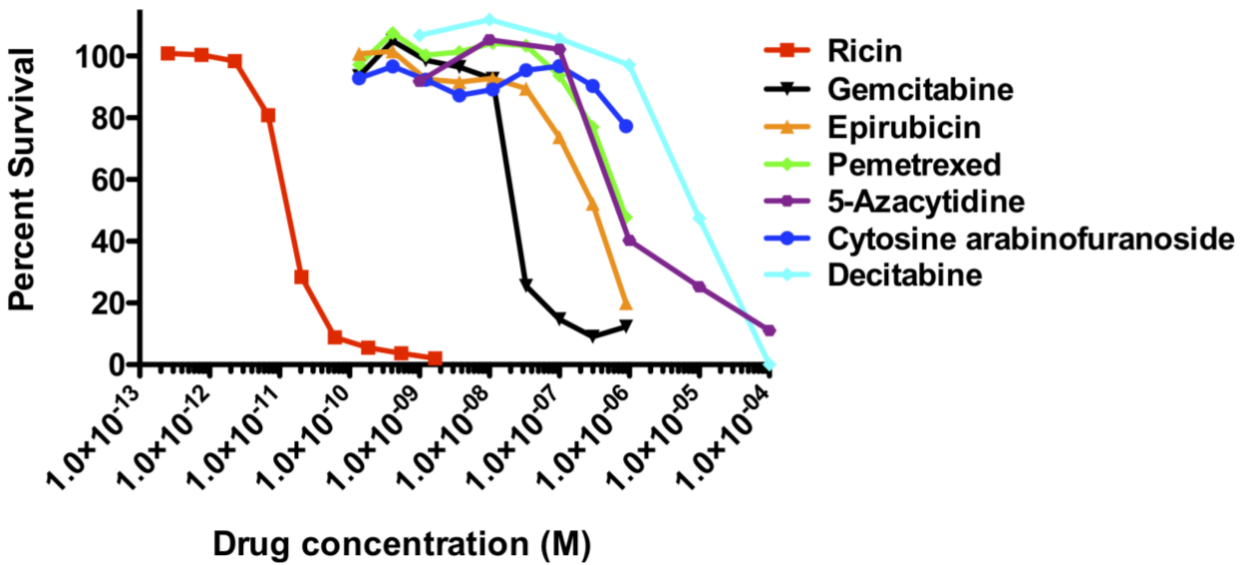
Comparative toxicity of ricin and chemotherapeutic agents on lymphoma cells. The comparative cytotoxic effect of chemotherapeutic agents and ricin was studied in the C8166 CD4+ lymphoma line using MTS dye reduction. On a molar basis, ricin was 4 logs more toxic than the smaller drugs.

When compared to cytotoxic agents used to treat cancer, RT is far more potent. Figure 2 shows agents that target CD4+ lymphoma cells. RT is 4 logs more potent (on a molar basis) than the most active cytotoxic drugs. Although RT has a relatively high molecular weight, MAbs armed with 1–2 molecules of RT retain antigen binding. For the smaller cytotoxic agents, that number is approximately 10 molecules bound to each MAb. Thus even by conjugating more cytotoxic drug per molecule of MAb, it is unlikely that a cytotoxic drug-conjugate will achieve the toxicity of the most potent RT/RTA-based ITs. We have coupled the same anti-HIV MAb to RTA and to doxyrubicin, and found excellent *in vitro* and *in vivo* killing with the RTA IT, but only non-specific killing with the doxyrubicin conjugate (C. Coyne and SHP, unpublished). 

Given the high degree of toxicity, as well as its general reputation as an agent of bioterrorism, it is no surprise that there have been concerns about the therapeutic use of RT. Considerable effort has gone into reducing the non-specific toxic side-effects of RTA-based ITs. VLS, hepatotoxicy, and nephrotoxicty were the major dose-limiting effects of very early ITs. It was subsequently found that mannose residues on the plant-derived RTA led to binding of RTA to mannose receptors on liver cells [31,32,33,34] and thus chemically or enzymatically deglycosylated RTA (dgRTA) was subsequently used. The molecular basis of VLS has been studied [35,36], and it may be reduced with anti-inflammatory drugs [37] or by altering the three amino acids in RTA that bind to endothelial cells and cause VLS. [36].

RTA is a proinflammatory and immunogenic molecule. RT and RTA can in rare cases be allergens. The presence of antibodies against RTA leads to rapid clearance of RTA-ITs from the blood, such that its efficacy is reduced. Hence, instead of a half-life of several days, it can be cleared in hours. However, if one waits 2–8 weeks, titers of antibody drop to baseline and further doses can be given. Several approaches have been taken to specifically suppress the development of anti-IT immune responses, including the concomitant administration of immunosuppressive agents such as cyclosporine, deoxyspergualin, anti-CD4 antibodies, or CTLA-4-IgG [38,39,40,41,42,43,44], or the development of tolerance with agents such as polyethylene glycol [45,46,47].

## 4. Ricin as a Weapon of Bioterrorism

RT is of concern in the area of biodefense because it is readily available to individuals or groups with little technical expertise or funding. Its source is a ubiquitous weed and a cultivated crop in many countries. Crude RT made from pulverized castor beans is toxic and a 2-step extraction with readily available chemicals yields >95% pure toxin. The toxin is chemically stable and can be stored unrefrigerated for long periods of time with little loss of activity. Over the past several decades, there have been well-documented instances of RT prepared for nefarious uses, and at least one well-documented assassination with RT [2,3,7]. Although some have underplayed the risk from RT, primarily because it is unlikely to cause mass casualties [7], we should not underestimate the panic and disruption that can result from a biological attack initiated by a lone lunatic, even if it only involves a small number of individuals. Public assurances that an antidote is at hand will greatly allay anxiety and act as a deterrent.

Measures designed to protect people from the lethal effects of RT will be different for civilian and military populations. For civilians, any one individual is at very low risk of exposure, but there is a high likelihood that there will eventually be an attack against the public that will likely involve a limited number of individuals. In this case, post-exposure treatment is most appropriate. In addition to developing appropriate therapies, effective use of post-exposure strategies will require recognition of the event by alert first responders, rapid confirmation of an attack *via* specific assays, obtaining the appropriate therapeutic agents, and administering them, all within a limited window of time (<24 h). Since the symptoms of RT poisoning do not appear for hours, and they are difficult to distinguish from many other common infections, this will be challenging. A factor that strongly mitigates the risk of exposure by a large population is the difficulty in delivering RT in uniformly lethal quantities. As discussed below, aerosol exposure is most likely to produce serious symptoms. Delivery of either dissolved toxin or milled powder requires particle size less than 3 microns [48], larger particles rapidly settle and cannot penetrate the pulmonary system as deeply. Particles < 1 micron may remain suspended as aerosols indefinitely. Hence some technical expertise would be needed to produce optimally weaponized aerosols, although this could be done by trained individuals working for an enemy group. As noted earlier, however, larger quantities of crude ricin could be effectively used as well. 

For the military, entire personnel units are likely to be exposed as a group. However, in this setting, RT has limited utility. The amount required for aerosol toxicity is large (10 µg/kg), compared to botulinum toxin (100 ng/kg), for example. Dispersion over a battlefield or military encampment is highly dependent upon weather conditions and technical expertise. Nevertheless, if the military were facing an enemy thought to have ricin in its arsenal, then pre-exposure preventative measures, which might include physical barriers, chemical detoxification agents, and immunization [6,49,50,51,52], should be planned.

## 5. Routes of Exposure

The route by which one is exposed to RT is a key determinant of the clinical symptoms observed. Military groups are most likely to be exposed *via* the aerosol route, whereas attacks against civilians could be by aerosol, ingestion, or possibly even injection. RT could be used in solution or in its powdered form. Even if aerosolized particles are not small enough to be fully inhaled into pulmonary spaces, damage to mucosal tissues is still possible. There are a number of unresolved issues regarding the clinical syndromes associated with RT exposure, among them: Does the oral route pose a significant risk to humans? Clearly the ingestion of castor beans can be fatal, but this might be due to the protective effect of the seed coat in the upper GI. It is not clear what the effect of pure toxin either in suspension or as a powder will be in humans. Several excellent reviews of the clinical effects of ricin have been written [2,3,4,5,7]. However, it should be emphasized that there are almost no human data regarding exposure to the pure toxin, so that the signs and symptoms attributed to RT exposure syndrome have, for the most part, been extrapolated from animal data.

### 5.1. Oral Exposure

The effects of ricin given orally vary depending upon whether purified toxin or castor beans are ingested, whether the toxin is administered by gavage feeding or orally, whether there is a full or empty stomach, and other factors. More than 1000 cases of human ingestion of castor beans have been reported. Release of toxin from the beans requires digestion and delipidation of the bean matrix, and thus the toxin is not released until it reaches the lower small intestine or the large bowel. Chewing the beans more thoroughly may increase toxin release. Symptoms attributable to the ingestion of castor beans occur primarily in the lower GI tract where RT can induce cramping, diarrhea, and blood in the stool. These can result in fluid and electrolyte imbalances. Even more serious sequelae, resulting from damage to the intestinal tissue, can occur. Mortality from castor bean ingestion may approach 2% [7]. 

All published studies of oral toxicity of RT have been performed in small animals, primarily rodents, and have utilized direct instillation of the toxin into the stomach (gavage feeding). Although gavage feeding allows for rapid and accurate dosing, it bypasses the oral mucosa and the esophagus, two sites where pathology and clinical findings may originate. Lethal doses for gavage feeding have generally been in the 15–35 mg/kg range, that is 1000× higher than the dose reported for aerosol or injection routes [53,54,55,56], although in mice fasted for 20 hours prior to gavage feeding, the lethal dose may be lowered 100 to 1000-fold [6]. At doses of 35 mg/kg, administered orally to mice, there was no clinical effect, no mortality, and only marginal pathological findings. (K. Song, S.H. Pincus, unpublished). Mortality was observed at 100 mg/kg. However, this involved feeding a mouse the human equivalent of 2 L of ricin at 25 mg/mL over 3 hours, and could have resulted in some degree of aspiration of ricin into the airways, where the lethal dose is 10,000-fold less. Withholding food before feeding ricin did not increase susceptibility. Thus it may be possible that purified RT, as compared to castor beans, has limited, if any, toxicity by the oral route.

It is not clear why purified RT administered by mouth is less toxic than when introduced by gavage directly into the stomach. It implies a degree of resistance of the oral and esophageal tissues to the toxic effects of RT. One possibility is that the microbial flora express carbohydrate structures with terminal galactose residues, and the microbial saccharides compete with host glycoproteins and glycolipids on tissue surfaces. Alternatively, a host saccharide in the saliva or other oral/esophageal secretions may be competing with tissues for ricin binding [96]. 

In any case, laboratory rodents may not be adequate as surrogates for studying oral exposure of humans, since their oral and esophageal mucosa differ markedly from those of humans. Because their herbivore diet largely consists of hard, fibrous material (mouse chow), the oral and esophageal mucosa of rodents maintains a luminal surface with a highly cornified layer of stratified epithelium, whereas in humans it is only minimally keratinized, and may be more easily damaged by direct action of the toxin. Entirely aside from the question of susceptibility of the upper GI tract to ricin-mediated damage, is the matter of taste. Castor oil, extracted from the castor bean and used as a purgative for many years, is notable for its terrible taste. The CDC states that ricin is odorless and tasteless (http://www.bt.cdc.gov/agent/ricin/clinicians/background.asp) but if the toxin is insufficiently pure and retains even a small portion of the taste of the castor oil, it may prove impossible to feed the requisite amount of toxin to cause toxicity. To address both of these issues, it is critically important to determine the oral toxicity of ricin in a non-human primate, whose esophageal mucosa is similar to that of humans and where taste aversion can be studied. If ricin is not palatable or non-toxic in these animals, then we can lower our level of concern regarding risks of oral exposure in human populations. 

### 5.2. Inhalation Exposure

Aerosolized RT is widely considered to be the most lethal route of exposure and certainly the one that has the potential to injure or kill the greatest number of victims in a terrorist attack. However, generating a fine aerosol that remains airborne at a sufficiently high concentration is technically more difficult than adding it to food or water. Most of the studies examining the toxicity of aerosolized RT have been performed in rodents but results are consistent with the limited data available from non-human primates [2,5]. In mice, aerosolized RT has an LD_50_ of 3–5 μg/kg. When it is inhaled, the size of the aerosol particle correlates inversely with the severity of lung damage, *i.e.* the smaller particles can penetrate more deeply into the lungs and cause more damage [2]. Most of the damage is observed within the lungs, which indicates that little toxin escapes from the mucosa. The resulting necrosis and inflammation lead to non-cardiogenic pulmonary edema and infiltration of immune cells. Subjects usually die of respiratory failure. Despite the localization of toxin in the lungs, systemic inflammation is still observed and can lead to arthralgias and fever. Understandably, it has been observed that pulmonary exposure to RT upregulates genes involved in inflammation and tissue remodeling, as well as the release of various cytokines and chemokines [56,57,58,59].

### 5.3. Injection of RT

RT injected intraperitoneally (i.p.), intramuscularly (i.m.), or subcutaneously (s.c.) has LD_50_s of 5 to 24 μg/kg. While this is nearly as lethal as inhaled aerosolized ricin, injections are not suitable for use as a “weapon” although RT has been used this way in the setting of espionage. The best documented case of this involved the 1978 assassination of Georgi Markov, a Bulgarian journalist and defector; a toxin-laden pellet was fired into his leg from the tip of an umbrella as he waited for a London bus. He died a few days later in hospital, since there was not (and still is not) a specific antidote for RT poisoning. 

As for the other routes, there are limited data in humans but they also compare well with animal data. Following injection, tissue necrosis is observed at the injection site (Markov reported immediate local pain). Within a few hours there is systemic inflammation, fever and hypotension, resulting in ‘flu like symptoms’ that mimic many other diseases. Systemically, injection results in severe local lymphoid necrosis, gastrointestinal hemorrhage, liver necrosis, diffuse nephritis, and diffuse splenitis. Alterations in liver transaminases, amylase, creatinine kinase, bilirubin, and myoglobin have also been reported following exposure. In mice, systemic injection of RT results in lethal hypoglycemia, with little evidence of other metabolic abnormality [60]. RT exerts its toxicity on many different cell types and so it may not be possible to pinpoint the exact cause of death. 

## 6. Protective Measures

Different populations at risk require distinct approaches to protection. For military units facing an enemy that is believed to have RT in its arsenal, immunization is the most logical approach. For civilians, where the likelihood of any one individual being exposed to the toxin is low, the emphasis needs to be placed on post-exposure treatment. Civilians will not accept the use of a vaccine unless there are continuing attacks. Unfortunately, post-treatment therapy requires early recognition of intoxication, rapid confirmation of the exposure, and easy access to specific treatment. This is difficult since the early symptoms can mimic those of many diseases including influenza. 

In this section we will discuss approaches to the prevention and treatment of RT toxicosis. Both specific and non-specific measures will be discussed. The authors of this article believe that antibodies currently offer the greatest potential for the development of specific RT inhibitors. Perhaps this is no surprise, since we are immunologists. This section will close with a discourse on passive and active antibody therapies. Active immunization will be of interest to the military and perhaps civilian first responders, whereas passive therapies are optimal for post-exposure treatment in any population.

### 6.1. Public Health Approaches to Containing Risk

Measures that assess and mitigate risks in populations can be applied to the matter of poisoning by RT or other toxins [2,3,7]. These measures would include an accurate depiction of the expected clinical syndrome. Of the likely routes of exposure, both the oral and parenteral routes lack a clear definition. Next, this information needs to be disseminated to those healthcare workers who are likely to encounter victims of an attack, so that they will consider RT poisoning in differential diagnosis. We also need appropriate diagnostic tools that allow the rapid and specific identification of intoxication by RT. This includes not only assays for detection as a white powder, but also assays that are validated for use in human body fluids. Immunoassays are most likely to be used for this purpose. 

Beyond this, population approaches diverge depending whether military forces or civilians are being considered. Critical to protecting the military is good intelligence. Foreknowledge of the presence of large stores of RT by one’s enemy would allow deployment of physical barriers, chemical decontamination procedures, stockpiling of useful therapeutic agents, and consideration of active immunization. We do not have access to military assessments and planning for such exposures.

Procedures for the protection of civilians have been the subject of discussion, resulting in the publication of guidelines for the response to a RT incident [3]. But key gaps do exist. Informatic tools to link doctor’s offices, hospital emergency rooms, and free standing “instant care” facilities to identify outbreaks in dispersed patients need to be developed [61]. Assays providing confirmation of the toxin’s presence in clinical samples have yet to be approved. Perhaps most worrisome of all, is that the critical steps and processes for recognition of an “RT incident” and steps to mitigate the effects are left to our overworked and underfunded state and local health departments, emergency rooms, and primary care facilities. It seems ironic that even as the government funds the development of new epidemiologic tools, diagnostics and treatments, we allow the crumbling of our primary bulwark against bioterrorism, the public health infrastructure.

### 6.2. Post-Exposure Therapies

Post-exposure treatments fall into two general categories, specific anti-RT agents and non-specific supportive therapies [2,3,4,5]. At the present time only the latter exist. RT-specific antibodies will be discussed in a separate section. Supportive therapies would include eliminating residual ricin from the body by exhaustive washing of affected mucosal or dermal tissues, and by gastrointestinal lavage. Maintaining proper fluid and electrolyte balance is critical. Following aerosol exposure, third-spacing of large volumes of fluid in the lungs occurs through the induction of inflammatory exudates, injury-caused transudation, and frank hemorrhage [48,62]. Similarly, diarrhea and hemorrhage associated with castor bean ingestion has been reported to cause substantial fluid loss. Following aerosol exposure, respiratory support may be necessary. This may include supplemental oxygen, continuous positive airway pressure breathing, and artificial ventilation. The use of pressor drugs to mitigate circulatory collapse may be required in advanced cases. Corticosteroids and therapies directed at inhibiting inflammatory cytokines may blunt the inflammation that creates much of the pathology, especially that seen following aerosol exposure [56,57,58,59].

If they existed, small molecule inhibitors of ricin toxicity might be used to treat patients who have been exposed to ricin. They may also be used prophylactically, if it were deemed likely that an exposure would occur. As yet, there are no specific ricin antidotes that have been approved for use, although several therapeutic targets are being explored in drug-discovery laboratories. These include agents that block the binding of RTB to cell-surface glycans [14], agents that block *N*-glycosidase activity [19,20,21,22,63], or specifically inhibit retrograde transport through the protein synthetic pathway [8]. Of these, only the inhibitors of retrograde transport have been tested in animals, and found to be somewhat effective in ameliorating ricin’s toxicity at acceptable doses. *In vitro* efficacy of the others is in the micromolar range, with varying degrees of non-specific cytotoxicity, and are unlikely to result in a therapeutic effect *in vivo*.

### 6.3. Active and Passive Immunization

As indicated earlier, the authors are proponents of immune therapies for RT toxicosis. Both a vaccine for active immunization [6,49,50,51,52] and antibodies for passive immunization [64,65,66] are under development. A vaccine is a pre-exposure preventive measure that is most likely to be utilized by the military, whereas passively administered antibodies can be used either as post-exposure treatment (civilians or military) or as prophylaxis for a very high-risk and short-term exposure (military).

#### 6.3.1. Passive Administration of Antibodies

Passive immunization predates the development of antibiotic therapies, circa WWII. Antibody therapy has seen a resurgence in recent years with the development of MAb technologies, protein engineering, and much improved biochemical techniques for the purification and formulation of immunoglobulin (Ig) preparations [67,68,69]. One of the few universally agreed upon truths of the field of vaccinology is that antibodies are the primary mode of protection from toxins. Vaccines that protect against bacterially-derived tetanus and diphtheria elicit protective antibodies against the toxin, and do not affect the organism directly. Passive antibody therapies to treat bacterial infections in which pathology is mediated by toxins, such as anthrax and pseudomembraneous colitis, have undergone clinical testing [70,71]. Thus there is great interest in how antibodies function to neutralize toxins. The general belief is that antibodies neutralize toxins by blocking their binding to target cells. Yet for many toxins, including RT, anti-RTA antibodies can be as effective as anti-RTB antibodies. This raises questions as to the mechanism by which anti-RTA antibodies neutralize RT. This could occur by preventing binding, internalization, or routing of the RTA to the endosomal compartment after the formation of immune complexes. Recent studies using quantitative confocal microscopy and cell kinetic experiments, clearly demonstrate that anti-RTA neutralizes by altering intracellular trafficking and by neutralizing the toxin inside the cell (S. Pincus and K. Song, submitted for publication). These studies also demonstrate that antibody can protect cells even when added 8 hours after the cells have been exposed to RT.

**Figure 3:**
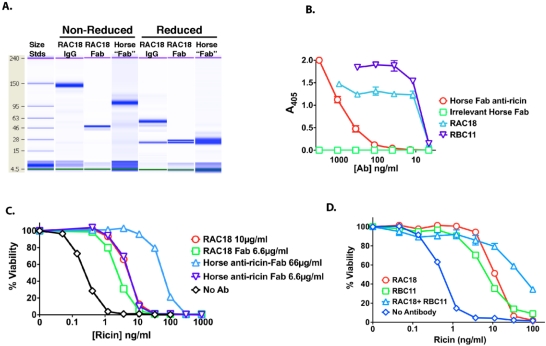
*In vitro* neutralization of ricin by polyclonal horse Fab preparations and MAbs. Panel A. Agilent microcapillary electrophoresis of intact monoclonal RTAs, MAb against RTA (RAC18 IgG *vs.* RAC18 Fab), and the polyclonal horse anti-ricin “Fab” preparation, under reducing and non-reducing conditions. The horse antibody preparation consists primarily of F(ab)’_2_ fragments. Panel B. ELISA binding of antibodies to plates coated with RT. Different secondary enzyme-conjugated antibodies were used to detect the horse and mouse Igs. Antibody concentration represents total protein. For MAbs, 100% is RT-specific antibody, whereas for the horse Fab, only 3.2% is RT specific. Panel C. *In vitro* neutralization of RT cytotoxicity by intact antibody and Fab fragments. Antibody concentration represents total protein. No antibody and control antibodies yield identical curves. Panel D. *In vitro* neutralization by high titer antibodies against RTA, (RAC), RTB (RBC), or both. Antibodies were used at 10 µg/mL.

The key question in defining the utility of passive antibody therapies is whether the antibody can have a protective effect in people when administered after a 12–24 hour delay, the minimum amount of time to recognize and confirm a RT exposure, and then to obtain and administer the antibody. Animal studies show *in vivo* protection by MAb RAC18 occurs even when administration is delayed by 12 hours [64,65]. Among other issues to be resolved are: which antibody to use; whether to target RTA, RTB or both; the use of polyclonal vs monoclonal antibody, intact antibody or antibody fragments; and whether systemic or local administration is better. Figure 3 provides data concerning some of these issues.

**Figure 4:**
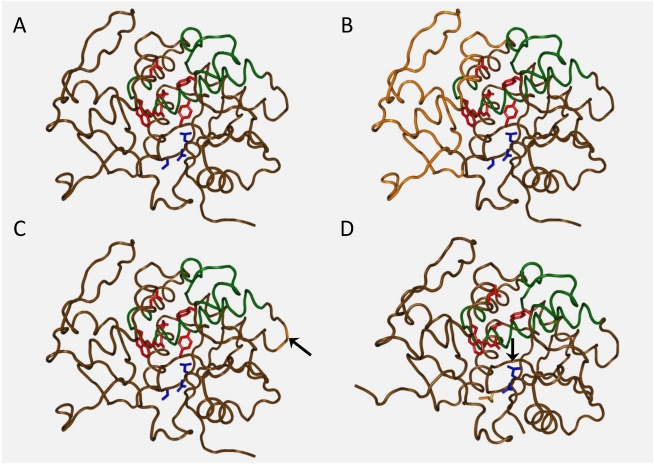
Crystal structure representations of the recombinant RTA vaccines. RTA active site residue side chains shown in red, VLS residue side chains (L74, D75 and V76) in blue, and dominant immunogenic epitopes in green. A. Wild type RTA; B. RTA1–33/44–198 (USAMRIID) vaccine: portions of the structure genetically excised shown in orange; C. Ricin-MPP (Warwick) vaccine: point of 25-mer insertion shown in orange (with arrow); D. RiVax (Texas) vaccine: Y80A V76M residue side chains shown in orange (Y80A has an arrow). (PDB accession No. 1RTC for panels A, B, and C, 3BJG for panel D.

A number of groups have evaluated different anti-RT MAbs for protection *in vitro*, *in vivo*, or both [18,66,72,73,74,75,76,77,78,79]. Unfortunately, most antibodies have not been compared side-by side. In our own studies with a panel of 38 different murine anti-RTA or RTB MAbs or to compound epitopes on both chains, we have found antibody affinity to be the most important determinant of *in vitro* protection ([66] and S. Pincus and K. Song, submitted). Epitope specificity may also play a role [66,78,79]. For *in vivo* protection, antibody isotype and Fc-mediated effects may also be important [80]. We, and others, have found that anti-RTA antibodies are generally more protective than anti-B chain antibodies *in vitro *(Figure 4, and reference [66], although others have reported the opposite [76]).

Figure 3 compares the efficacy of a polyclonal Fab preparation to that of our best anti-RTA and RTB MAbs, RAC18 and RBC11, respectively. The polyclonal antibodies were elicited by immunization of horses with an RTA/RTB chain construct, in which the native inter-chain linking domain has been replaced by an uncleavable linker. The animals were hyperimmunized and then repeatedly bled. IgG was purified from the plasma, and a mixture of Fab and F(ab)’_2_ fragments were prepared by proteolytic digestion (Figure 3A). Most of the mixture consisted of F(ab)’_2_. Because the Fc, a site of many species-specific epitopes, has been removed, these Abs are termed “despeciated” with the expectation of reduced immunogenicity. Concentration of RT- binding antibody was 32 µg per mg of total protein, as determined by Biacore analysis under conditions of partial mass transport limitation [81]. RT binding by the equine Fab preparation was markedly less than that of RAC18 and RBC11 as determined by ELISA (Figure 3B), even when recognizing that only 3.2% of the polyclonal preparation is RT-specific Fab. The reverse occurred when antibody-mediated neutralization was determined (Figure 3C). The equine Fab was 30X more potent than the best MAb, RAC18. Equimolar concentrations of horse Fab (6.6 µg/mL) and intact RAC18 have inhibition curves that completely overlap, yet only a small fraction (3.2%) of the polyclonal preparation is RT-specific. Figure 3C also demonstrates that a Fab fragment derived from RAC18 has marginally lower neutralization activity than the intact antibody. Figure 3D shows greater neutralization by anti-RTA than by anti-RTB MAb. It also shows that additive effects can be obtained when the two are mixed. Together these results demonstrate that Fab fragments can neutralize toxin *in vitro*, and that there may be advantages to polyclonal preparations.

The development and utilization pathway for anti-RT antibodies is fairly straightforward. A central agency must ultimately choose one optimal antibody formulation. Ultimately, this will be determined by demonstrating protection in a non-human primate aerosol challenge model. The best efficacy would be determined after a delay of 12–24 hours following exposure to the toxin. Formulations of the antibody would be stockpiled at locations that are sufficiently dispersed to accomplish delivery to any exposed individual. Point of care diagnostic tests and standards must also be established. Health care workers must know how to order antibodies from central stockpiles capable of providing service within hours. Of course, the majority of these steps also apply to many other infectious health threats. 

#### 6.3.2. Active Immunization

As discussed above, active vaccination is probably not suitable for the public at large, but would be useful for military personnel, who might be intentionally targeted, as well as emergency first responders, who are the most likely to be exposed domestically, either accidentally during an investigation of an individual in possession of small amounts, or during a terrorist attack. Only if RT were used repeatedly in domestic terrorist attacks would the public be vaccinated. As such, the availability of effective countermeasures, including post-exposure therapies and a stockpile of an active vaccine, might serve as a deterrent. The ideal vaccine would protect against ricin exposures by any route, but in particular a mucosal route, since this would be the most likely route of exposure. For large emergency immunization campaigns, the vaccine should have a long shelf life and induce the formation of long-lived protective antibodies after 1 or 2 doses.

Several approaches to actively vaccinate against RT have been investigated. The most straightforward approach to generating a vaccine against any toxin is to irreversibly convert it into an inactive toxoid using heat or chemicals. However this process must preserve the key epitopes necessary for immunization. Ricin toxoid has been extensively studied in rodent models, both as subcutaneous (s.c.) injections and as various microsphere formulations administered mucosally [82,83,84,85]. It was effective in preventing death but did not prevent lung damage following aerosol or intratracheal exposures. Toxoid administered orally, alone or as a microsphere formulation, showed only minimal protection against inhaled RT. However, the toxoid has or can regain residual toxicity and has been considered too risky for a vaccine. 

An alternative to ricin toxoid is to vaccinate with one of its two subunits, RTA or RTB, which are orders of magnitude less toxic than the holotoxin. Deglycosolated (dg) RTA has been evaluated for use as a vaccine. Deglycosylation prevents liver uptake and therefore liver damage [86,87]. Most of the studies using dgRTA were concerned with generating a good mucosal response, apparently in the belief that this was absolutely required for protection against a mucosal challenge. However, as discussed below, we and others have developed recombinant RTA vaccines that, when given systemically, protect mice against aerosolized and orally administered ricin. Hence there is no absolute requirement for local sIgA production. Various dgRTA formulations with or without mucosal adjuvants were tested in rodents but none induced titers comparable to, or protected as well as, mucosally administered toxoid [88,89]. This lack of success, combined with concerns that it is too toxic for human use, even though it approximately three logs less toxic than RT, has resulted in it being dropped from consideration. Instead three different laboratories have chosen the safer approach of developing recombinant RTA vaccines. As shown in Figure 4, each utilizes a different strategy to eliminate the cytotoxic activity while maintaining immunogenic epitopes critical for inducing protective neutralizing antibodies [49,90,91].

As noted previously [49,90,91], one recombinant RTA consisted of the RTA subunit containing a 25 amino acid fragment to disrupt the enzymatic active site (Figure 4B). The rationale for this was based upon a precursor state of homologous proteins found in plants. In these plants a Type III ribosome inactivating protein (RIP) is produced as a zymogen that has an amino acid insert which interferes with the active site until it is converted to the active state by post-translational excision of this fragment. Rats vaccinated with this construct were protected against ricin delivered via the intratracheal route. While immunogenic and protective in animals, residual catalytic activity made it an unlikely candidate for a human vaccine [90]. It is not known at this time whether this vaccine is undergoing further development. 

Another strategy was to genetically eliminate the entire hydrophobic face of RTA that is normally shielded by RTB in the holotoxin, in an effort to increase solubility and stability. This truncated molecule, RTA 1-33/44-198 (Figure 4C), was more stable and less prone to aggregation during long term storage. This deletion also removed a portion of the active site and so this molecule also lacks enzymatic activity. This truncated subunit vaccine still retains known important immunogenic peptides, however the large deleted portion may contain other unidentified but important protective epitopes. This vaccine did not induce VLS activity *in vitro* [92] and performed very well in mice challenged with either injected or aerosolized ricin. However, lung function following aerosol challenge was not reported. The vaccine was effective in non-human primates (Leonard Smith, personal communication) and has been shown to be immunogenic and non-toxic in rabbits in a pre-clinical toxicology study [93].

In view of our discovery that dgRTA-containing ITs induce VLS via a (x)D(y) motif in RTA [35,36], we chose to engineer RTA to modify both the *N*-glycosidase and the VLS sites. Two point mutations, one in each of the two sites (Figure 4D), were introduced into the structural gene to generate a non-toxic highly immunogenic vaccine to protect mice against ricin [49]. The crystal structure of this construct, Y80A V76M (RiVax) was nearly identical to the wild type RTA, suggesting that the majority of the conformational epitopes should be intact [94]. RiVax has proven to be highly soluble and stable in a variety of formulations. Administered i.m., it is protective in mice in each of the three challenge models we have tested, oral gavage, injected or inhaled aerosol [6]. In the aerosol model, lung function tests and histological examinations at intervals post-exposure demonstrated that RiVax protected the mouse lungs in a dose dependent manner. This is important since the vaccine must also protect against debilitating damage, even if it is reversible, which appears to be the case. Since it is generally believed that i.m. vaccination does not induce mucosal secretory immunoglobulin A(sIgA) responses, we hypothesize that high titers of serum IgG antibody are sufficient to protect mice from a mucosal challenge with RT. This was confirmed by Neal, *et al*. [95], who immunized mice lacking secretory IgA using RiVax/alum administered s.c. and found that they were protected against RT administered by gavage, further substantiating our findings and our hypothesis. As compared to administration of RiVax administered i.m. when RiVax was given *via* the intradermal (i.d.) route we found a marginal improvement in its ability to induce protective antibody titers [52]. This vaccination route would be ideal for situations where rapid immunization of large groups was required. We have also completed one clinical trial of the vaccine (without adjuvant) in humans and have found the vaccine to be safe and immunogenic [51]. A second trial using an alum formulation is ongoing. This vaccine has been out-licensed to Soligenix for more advanced clinical trials and (hopefully) eventual FDA approval as an orphan drug for military personnel.

## 7. Future Considerations

Since several passive and active vaccines appear promising, it is important that the best of these reach the national stockpile for future use. Because RT is considered by many not to be a high priority biothreat the funding needed to accomplish this is difficult to obtain. Considering the efforts and resources already spent developing RT vaccines and antibodies, and the relative certainty that an incident will occur in the future, we view this as short-sighted and hope that government officials will rethink this policy.
